# Ovarian Cancer

**DOI:** 10.1155/2014/764323

**Published:** 2014-12-21

**Authors:** Yong Sang Song, Hee Seung Kim, Daisuke Aoki, Danny N. Dhanasekaran, Benjamin K. Tsang

**Affiliations:** ^1^Department of Obstetrics and Gynecology, Seoul National University College of Medicine, Seoul 110-744, Republic of Korea; ^2^Cancer Research Institute, Seoul National University College of Medicine, Seoul 110-799, Republic of Korea; ^3^Department of Obstetrics and Gynecology, School of Medicine, Keio University, Tokyo 160-8582, Japan; ^4^Department of Cell Biology, The University of Oklahoma Health Sciences Center, Oklahoma City, OK 73104, USA; ^5^Department of Obstetrics and Gynecology and Department of Cellular and Molecular Medicine, University of Ottawa, Ottawa, ON, Canada K1Y 4E9

Ovarian cancer is the most lethal gynecologic malignancy and is the seventh leading cause of cancer deaths in women worldwide. Despite advances in surgery and chemotherapy, overall cure rate has remained very low. The poor clinical outcomes mainly come from the high percentage of cases being diagnosed at an advanced stage disease due to the lack of effective screening methods and frequent emergence of chemoresistance. Recent evidences have suggested that cancer stem cells may also contribute to the development of chemoresistance. However, there are still many questions on ovarian carcinogenesis and mechanisms of chemoresistance of ovarian cancer, which need to be resolved to improve the treatment outcomes of ovarian cancer.

The research topics include molecular genetics of ovarian carcinogenesis, autophagic reaction in ovarian cancer, chemoprevention using phytochemicals, tumor heterogeneity issue, and dual carcinogenesis of the ovary (type I versus II). The paper entitled “*BRCA-associated ovarian cancer: from molecular genetics to risk management*” by G. Girolimetti et al. demonstrated that ovarian cancer arising in BRCA 1 or 2 mutation carriers may have peculiar molecular, pathological, and clinical features. They also suggested that BRCA 1 or 2 mutational analyses would be helpful in tailoring ovarian cancer management based on BRCA status in the future.

The work by G. Valente et al. showed that the positive expression of BECLIN 1 with a crucial role in the regulation of both autophagy and cell death was correlated with the presence of LC 3 positive autophagic vacuoles and was inversely correlated with the expression of BCL-2 inhibiting the autophagy function of BECLIN 1. They also suggested that a low level of autophagy might favor cancer progression and that ovarian cancer with upregulated autophagy might have a less aggressive behavior and be more responsive to chemotherapy in the paper entitled “*Expression and clinical significance of the autophagy proteins BECLIN 1 and LC3 in ovarian cancer*.”

The work by V. D. Martinez et al. assessed DNA copy-number loss (CNL), promoter hypermethylation, mRNA expression, and sequence mutation of KEAP1/CUL3/RBX1 complex as a regulator of the NFE2-related factor 2 (NRF2) pathway initiating response to oxidative stress in a cohort of 568 serous ovarian carcinomas form The Cancer Genome Atlas. They suggested that a remarkably high frequency of DNA and mRNA alterations may affect components of the KEAP1/CUL3/RBX1 complex, through a unique pattern of genetic mechanisms in the paper entitled “*Unique pattern of component gene disruption in the NRF2 inhibitor KEAP1/CUL3/RBX1 E3-ubiquitin ligase complex in serous ovarian cancer*.”

The paper entitled “*Phytochemicals: a multitargeted approach to gynecologic cancer therapy*” by L. Farrand et al. demonstrated molecular mechanisms of phytochemical action in cancer prevention and phytochemical-based approaches to overcome chemoresistance and phytochemical analogues and chemical modifications for greater efficacy. They suggested that high-throughput screening methods, rational modification, and developments in regulatory policies would accelerate the development of novel therapeutics based on phytochemical compounds, which would likely improve overall survival and quality of life for patients with gynecologic cancers.

C. Yuan et al. performed a meta-analysis to examine whether the* XRCC3* polymorphisms are associated with ovarian cancer risk in the paper entitled “*Analyzing association of the XRCC3 gene polymorphism with ovarian cancer risk*.” They found no association between XRCC3 rs861539 polymorphisms and ovarian cancer, whereas they observed a significant correlation with ovarian cancer risk using the homozygote comparison (T2T2 versus T1T1), heterozygote comparison (T1T2 versus T1T1), and the recessive genetic model (T2T2 versus T1T1 + T1T2). For XRCC3 rs1799796 polymorphisms, they also found a significant correlation with ovarian cancer risk using the heterozygote comparison (T1T2 versus T1T1).

G. Shuvayeva et al. demonstrated that single amino acid arginine deprivation triggered profound prosurvival autophagic response in cultured human ovarian cancer SKOV3 cells in the paper entitled “*Single amino acid arginine deprivation triggers prosurvival autophagic response in ovarian carcinoma SKOV3*.” They also found that a significant drop in viability of arginine-starved SKOV3 cells was observed when autophagy was inhibited by either coadministration of chloroquine or transcriptional silencing of the essential autophagy protein BECLIN 1, suggesting that arginine deprivation-based combinational treatments that include autophagy inhibitors may produce a stronger anticancer effect as a second line therapy for a subset of chemoresistance ovarian cancers.

The work by R. Titone et al. demonstrated that the mRNAs of several autophagy-related genes contain the target sequence for miRNAs belonging to different families with either oncosuppressive or oncogenic activities in the paper entitled “*Epigenetic control of autophagy by microRNAs in ovarian cancer*.” Furthermore, they emphasized that plasma and stroma-cell derived miRNAs in tumor-bearing patients could impact autophagy.

The work by M. Koshiyama et al. mentioned a recent theory of dual carcinogenesis of the ovary in the paper entitled “*Recent concepts of ovarian carcinogenesis: type I and type II*.” In this review, they demonstrated that low grade serous carcinomas may be thought to evolve in a stepwise fashion from benign serous cystadenoma to a serous borderline tumor while the serous tubal intraepithelial carcinomas of the junction of the fallopian tube epithelium with the mesothelium of the tubal serous undergo malignant transformation to high grade serous carcinomas due to their location and metastasize to the nearby ovary and surrounding pelvic peritoneum.

The paper entitled “*Application of microRNA in diagnosis and treatment of ovarian cancer*” by K. Banno et al. suggested that many miRNAs have altered expression in ovarian cancer compared to normal ovarian tissues and these changes may be useful for diagnosis and treatment. Thus, they expect that chemotherapy targeting epigenetic mechanisms associated with miRNAs may also be effective to reverse gene silencing.

The paper entitled “*Expression profiles of epithelial-mesenchymal transition-associated proteins in epithelial ovarian carcinoma*” by M.-K. Kim et al. investigated the expression of Snail and Slug, the key regulators of epithelial-mesenchymal transition (EMT), in the primary ovarian cancer samples to assess the clinical significance of EMT-associated proteins. They found that Snail was differentially expressed according to the histologic subtype and was predominantly expressed in serous and endometrioid types. In the serous and endometrioid adenocarcinomas, the expression of Snail remained high across the stage and grade, suggesting its role in the early phase of carcinogenesis.

S. Mehrabi et al. assessed the levels of oxidative modified proteins in 100 primary serous epithelial ovarian carcinomas and normal/surrounding tissues using spectrophotometric, dinitrophenylhydrazone (DNPH) assay, two-dimensional gel electrophoresis, and Western blot analyses in the paper entitled “*Oxidatively modified proteins in the serous subtype of ovarian carcinoma*.” They showed that the levels of reactive protein carbonyl groups increased as stages progressed to malignancy, and the levels of protein carbonyls in serous ovarian carcinoma among African Americans are 40% higher reactive to Caucasian at similar advanced stages.

In summary, molecular genetics and autophagic reaction in ovarian carcinogenesis, multitargeted approaches using autophagic reaction and phytochemicals, and dual approaches considering types I and II ovarian carcinogenesis are of paramount importance. This special issue presents new perspectives on carcinogenesis and chemoresistance of ovarian cancer, which will be helpful in overcoming the limitations of diagnosis and treatment of ovarian cancer in the future.

*Yong Sang Song* *Hee Seung Kim* *Daisuke Aoki* *Danny N. Dhanasekaran* *Benjamin K. Tsang*

